# Supplementary material for: Combinatorial regulation of the balance between dynein microtubule end accumulation and initiation of directed motility
Source: EMBO J. 2017 Oct 16;36(22):3387–404. doi: 10.15252/embj.201797077 (PMC5686545; doi:10.15252/embj.201797077)
Supplement: Supplementary file 1 — Expanded View Figures PDF [file EMBJ-36-3387-s001.pdf]

Expanded View Figures

A HeLa cell dynactin

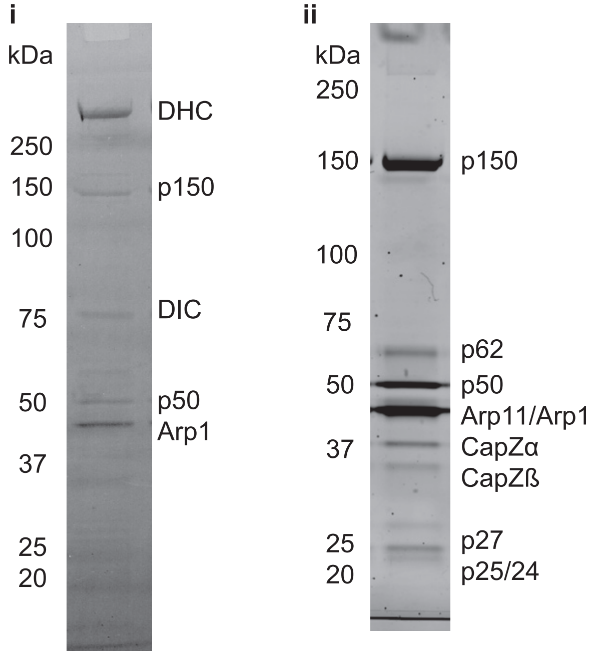

iii

Dynactin subunits	NCBI accession number	Unique peptide count	Sequence coverage (%)
**p150**	A8MWX9_HUMAN	58	46
**p50**	DCTN2_HUMAN	21	52
**p62**	DCTN4_HUMAN	11	21
**p24**	DCTN3_HUMAN	11	45
**p25**	DCTN5_HUMAN	8	42
**p27**	DCTN6_HUMAN	2	9
**Arp1α**	ACTZ_HUMAN	17	47
**Arp1β**	ACTY_HUMAN	7	43
**Arp11**	ARP10_HUMAN	18	34
**β actin**	ACTB_HUMAN	16	42
**CapZβ**	B2R7T8_HUMAN	16	48
**CapZα**	CAZA1_HUMAN	12	35
**Dynein subunits**	**NCBI accession number**	**Unique peptide count**	**Sequence coverage (%)**
**DHC1**	DYHC1_HUMAN	11	2
**DHC2**	DC1I2_HUMAN	13	21

B

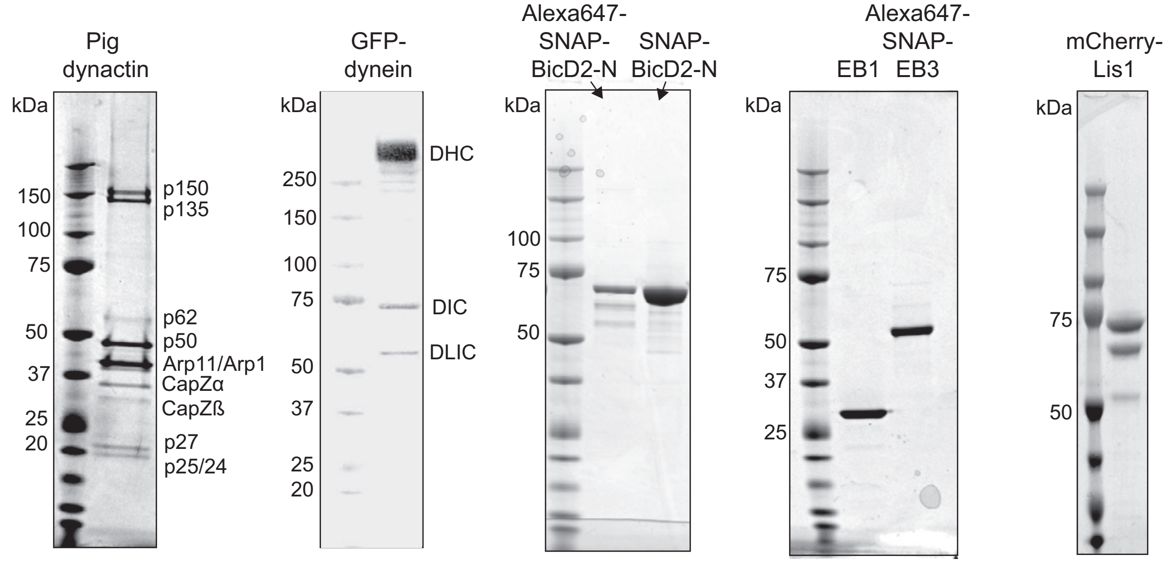

Figure EV1.

© 2017 The Francis Crick Institute Limited

◀ **Figure EV1. Purified proteins used in this study (related to Fig 1).**

A Purification of human dynactin from HeLa S3 cells: (i) Coomassie Blue-stained SDS–PAGE of the eluate from a BicD2 affinity column (first chromatography step of the dynactin purification). Bands corresponding to the dynein and dynactin complex are labelled. (ii) Sypro Ruby-stained SDS–PAGE of the pooled peak fractions of purified dynactin after anion exchange chromatography. (iii) Mass spectrometry results of the purified human dynactin complex demonstrating the presence of all subunits of the dynactin complex; some fragments of the dynein heavy and intermediate chains, but not of the smaller dynein subunits were also detected.

B Coomassie Blue-stained SDS–PAGE of the purified proteins used in this study, as indicated. The individual dynactin and dynein subunits are labelled. (The double band of mCherry-Lis1 originates from different mCherry maturation states, as previously observed for other mCherry tagged proteins; Duellberg *et al*, 2014.)

Source data are available online for this figure.

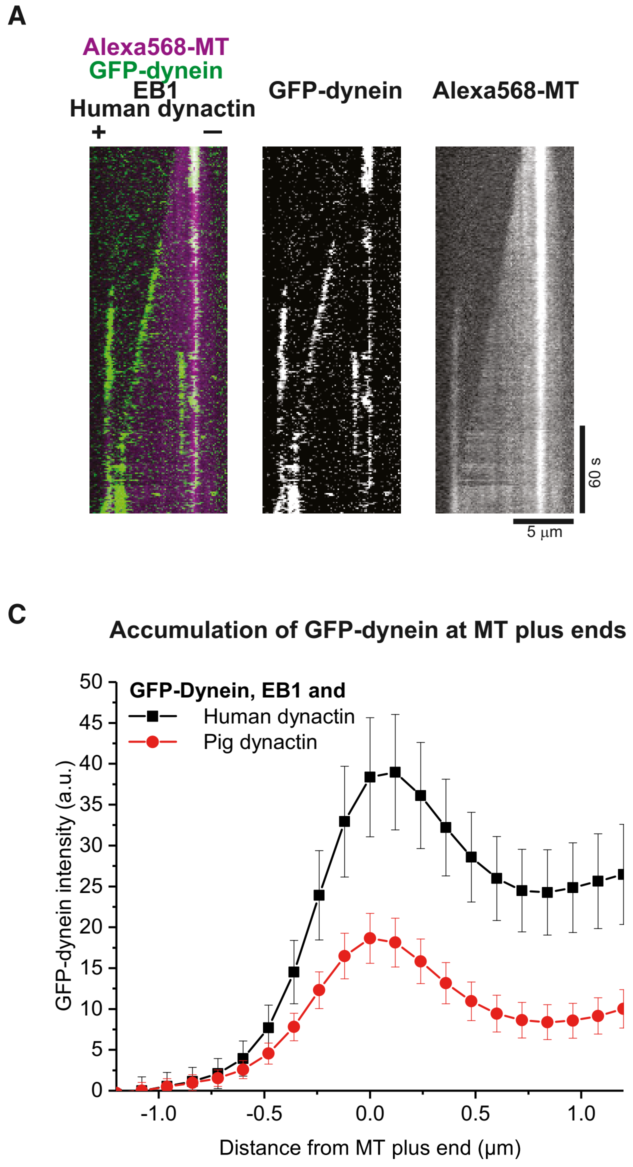

Figure EV2. Microtubule end tracking of GFP-dynein in the presence of EB1, and either pig or human dynactin (related to Fig 1).

A, B Dual colour kymographs of GFP-dynein (green) tracking the ends of Alexa568-microtubules (magenta in merge) in the presence of EB1 and either pig dynactin (A) or human dynactin (B). Protein concentrations were 10 nM GFP-dynein, 10 nM pig or human dynactin, 20 nM EB1 and 17.5 µM tubulin.

C Averaged fluorescence intensity profiles of GFP-dynein at growing microtubule plus ends in the presence of EB1 and either human (black circle), or pig (red circles) dynactin. Mean values from at least two separate experiments per condition (with a total of at least 47 kymographs) are shown; error bars are s.e.m.

Data information: Experiments were performed at 30°C.

 © 2017 The Francis Crick Institute Limited

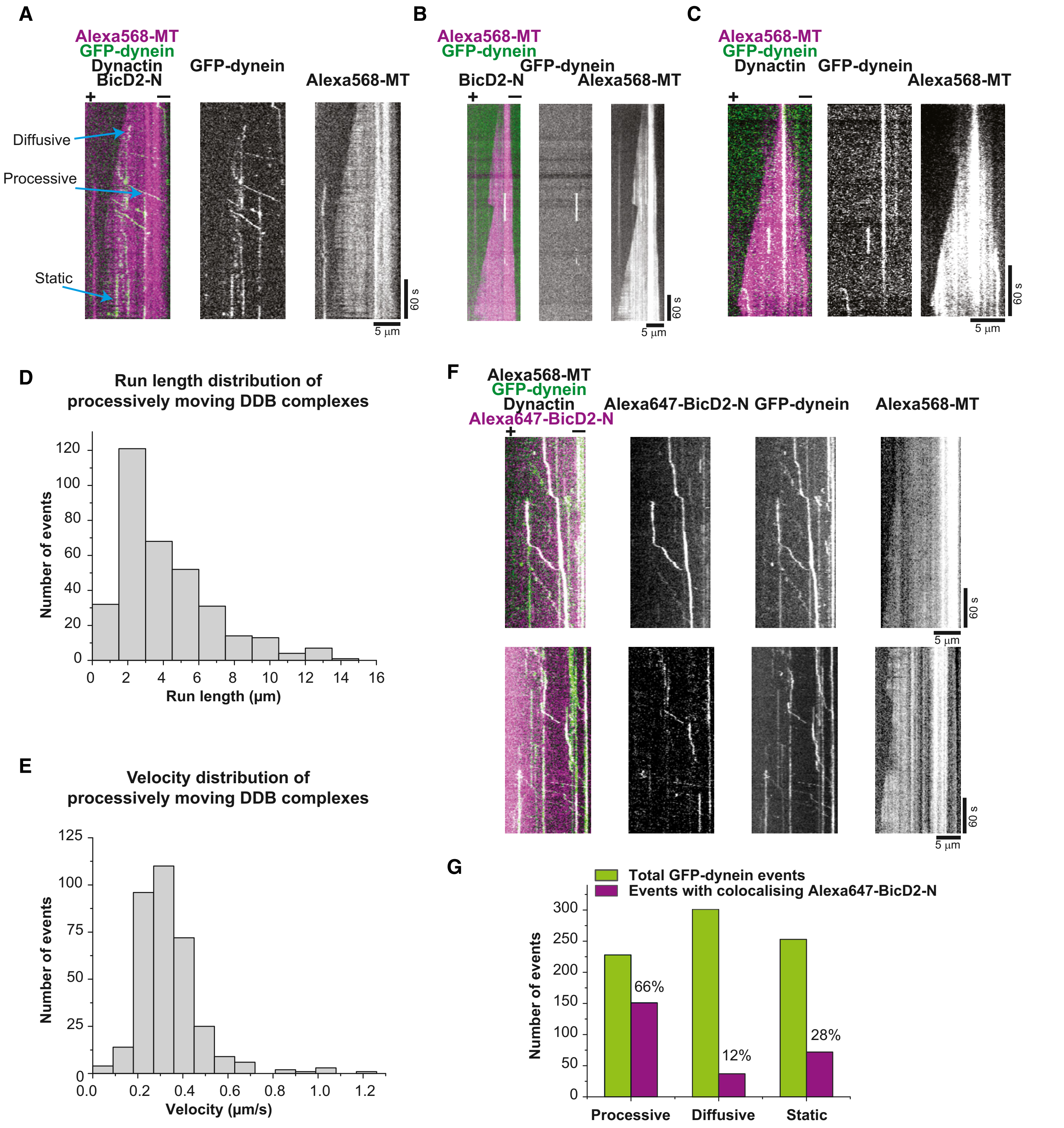
A
Alexa568-MT
GFP-dynein
Dynactin
BicD2-N
GFP-dynein
Alexa568-MT
Diffusive
Processive
Static
60 s
5 µm
B
Alexa568-MT
GFP-dynein
BicD2-N
GFP-dynein
Alexa568-MT
60 s
5 µm
C
Alexa568-MT
GFP-dynein
Dynactin
GFP-dynein
Alexa568-MT
60 s
5 µm
D
Run length distribution of processively moving DDB complexes
Number of events
Run length (µm)
E
Velocity distribution of processively moving DDB complexes
Number of events
Velocity (µm/s)
F
Alexa568-MT
GFP-dynein
Dynactin
Alexa647-BicD2-N
Alexa647-BicD2-N
GFP-dynein
Alexa568-MT
60 s
5 µm
G
Total GFP-dynein events
Events with colocalising Alexa647-BicD2-N
Number of events
66%
12%
28%
Processive
Diffusive
Static

Figure EV3.

Figure EV3. Dynein motility in the presence of dynactin and BicD2-N on dynamic microtubules (related to Fig 2).

A Dual and single colour TIRF microscopy kymographs depicting GFP-dynein motility (green in merge) on dynamic Alexa568-microtubules (magenta in merge). Concentrations were 10 nM GFP-dynein, 20 nM dynactin, 200 nM BicD2-N and 17.5 μM Alexa568-tubulin.

B, C Kymographs showing GFP-dynein behaviour in the absence of (B) BicD2-N or (C) dynactin; other conditions as in (A).

D Histogram of the run length distribution of processively moving DDB complexes (n = 343 complexes from three separate experiments). The mean run length is 4.1 ± 0.15 (s.e.m.) μm.

E Histogram of the velocity distribution of processively moving DDB complexes (n = 343 complexes). The mean velocity is 0.34 ± 0.01 (s.e.m.) μm/s.

F Dual and single colour kymographs showing preferential colocalisation of GFP-dynein (green in merge) and Alexa647-BicD2-N (magenta in merge) during processive motility in the presence of dynactin on dynamic Alexa568-microtubules (not shown in merge). Protein concentrations were 10 nM GFP-dynein, 20 nM human dynactin, 200 nM Alexa647-BicD2 and 17.5 μM Alexa568-tubulin. Microtubule orientation as indicated.

G Quantification of the number of observed GFP-dynein (green bars) and Alexa647-BicD2-N (magenta bars) events in the three categories "processive motility", "diffusion" and "static binding" and the percentage of events with colocalising BicD2-N in each category. Protein concentrations as in (F). Most colocalisation was observed for processive motility events (n = 788 complexes from two separate experiments).

Data information: Experiments were performed at 30°C.

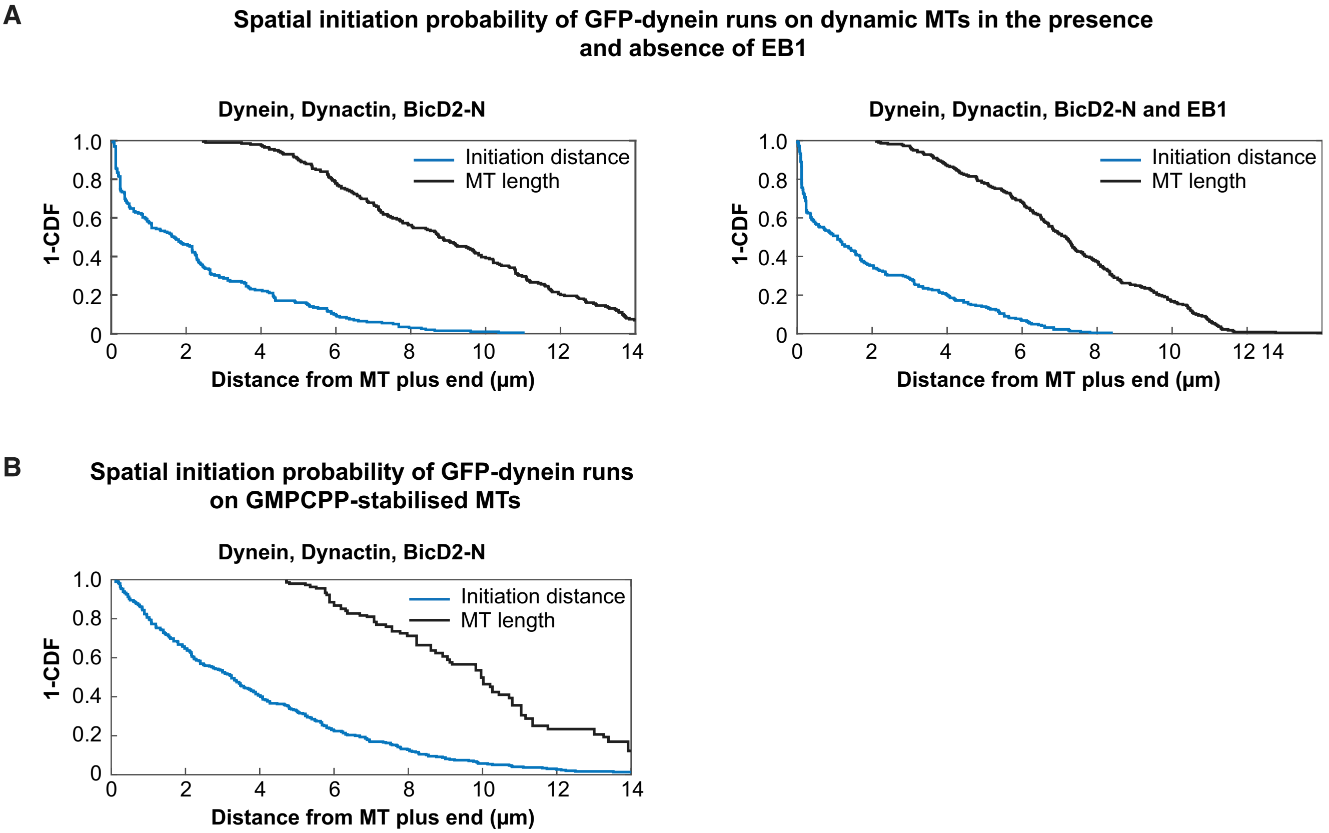

Figure EV4. Distributions of initiation probabilities and microtubule lengths (related to Fig 3).

"1 – cumulative probability" distribution functions of the distances of processive run initiation, measured from the microtubule plus end (blue), and "1 – cumulative probability" distribution function of the corresponding microtubule lengths (black) measured at each moment of run initiation.

A Conditions as shown in Fig 3B, black and red.

B Conditions as shown in Fig 3D.

 © 2017 The Francis Crick Institute Limited

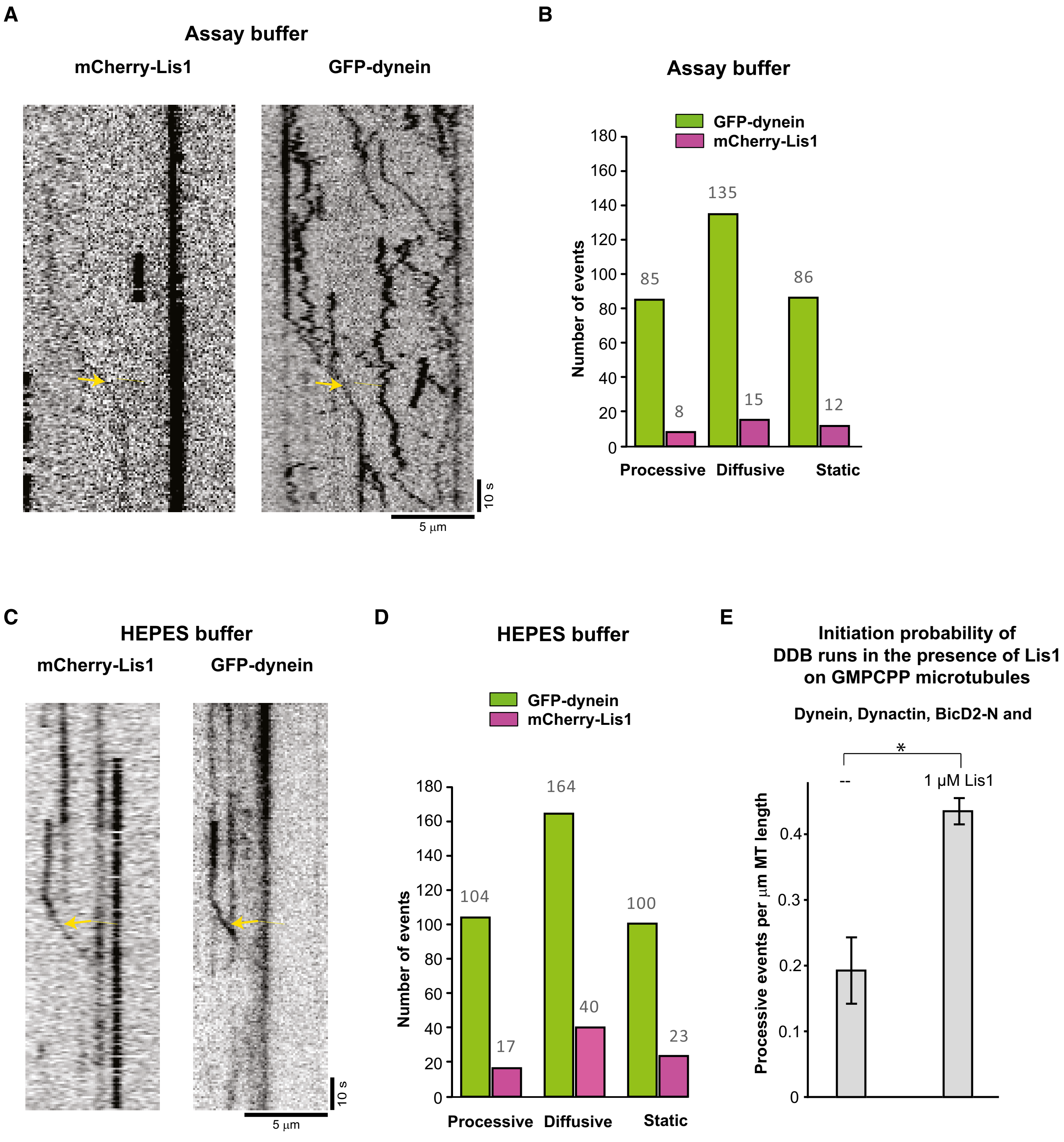
A
Assay buffer
mCherry-Lis1
GFP-dynein
10 s
5 µm
B
Assay buffer
GFP-dynein
mCherry-Lis1
Number of events
180
160
140
120
100
80
60
40
20
0
85
8
135
15
86
12
Processive
Diffusive
Static
C
HEPES buffer
mCherry-Lis1
GFP-dynein
10 s
5 µm
D
HEPES buffer
GFP-dynein
mCherry-Lis1
Number of events
104
17
164
40
100
23
Processive
Diffusive
Static
E
Initiation probability of
DDB runs in the presence of Lis1
on GMPCPP microtubules
Dynein, Dynactin, BicD2-N and
*
--
1 µM Lis1
Processive events per µm MT length
0.4
0.3
0.2
0.1
0

Figure EV5.

Figure EV5. Lis1 and DDB on static microtubules (related to Fig 5).

A–D Colocalisation of Lis1 with DDB particles. (A) Single-channel TIRF microscopy kymographs (inverted LUT) depicting GFP-dynein and mCherry-Lis1 on GMPCPP stabilised microtubules in the presence of dynactin, BicD2-N in assay buffer AB (as used for almost all experiments in this study). Arrows indicate an event where Lis1 colocalises with dynein. (B) Quantification of the total number of observed processive, diffusive and static GFP-dynein events (green bars) and those with colocalising mCherry-Lis1 (magenta bars) in assay buffer AB. 9% of the processive DDB particles were observed to have Lis1 bound. (C, D) Corresponding data (as in A, B) for control experiments in a different HEPES-based buffer (see Materials and Methods). 16% of the processive DDB particles were observed to have Lis1 bound. Concentrations were 5 nM GFP-dynein, 10 nM pig dynactin, 200 nM BicD2-N and 50 nM mCherry-Lis1; 306 and 368 DDB particles were analysed for Assay Buffer and HEPES-based buffer, respectively (from two experiments each).

E Effect of Lis1 on the initiation of processive DDB runs. Bar graph showing the average number of processive DDB runs per micrometre of GMPCPP-stabilised microtubule. Protein concentrations were 5 nM GFP-dynein, 10 nM pig dynactin, 200 nM BicD2-N and either no Lis1 (left bar) or 1 μM Lis1 (right bar). Error bars are s.d. $*P = 0.04$ (unpaired *t*-test). For DDB alone, 37 complexes were analysed, and for DDB in the presence of Lis1, 87 DDB complexes were analysed, each from two different experiments.

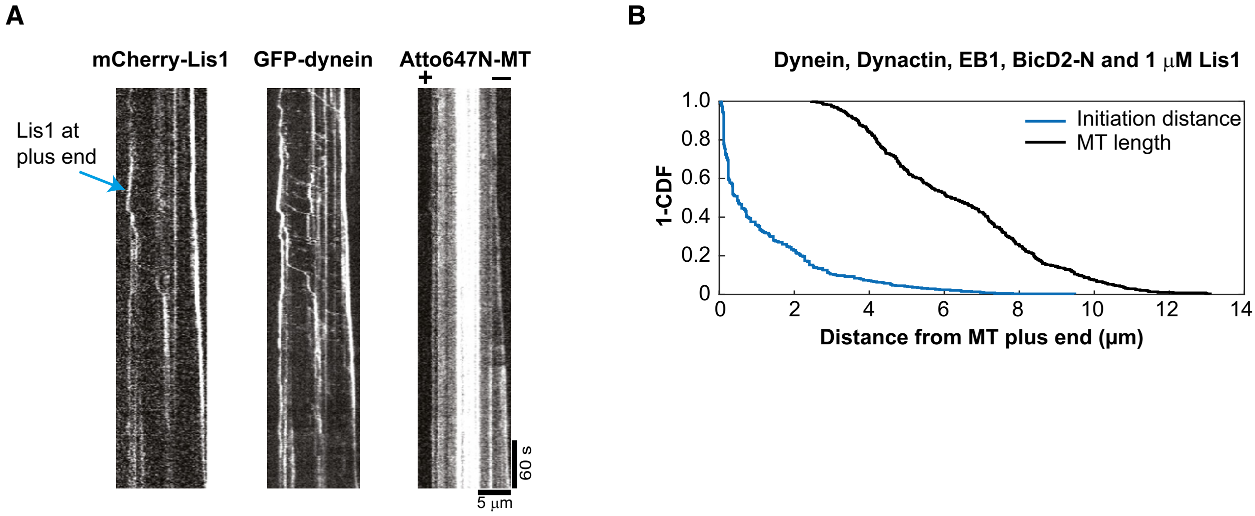

Figure EV6. Spatial initiation probability of DDB runs within the first 7 μm from growing microtubule plus ends in the presence of EB1 and Lis1 (related to Fig 5).

A Single colour kymographs of a triple colour TIRF microscopy experiment showing mCherry-Lis1 colocalising with GFP-dynein at dynamic Atto647N-microtubule plus ends. Protein concentrations were as in Fig 5B. Microtubule orientation as indicated. Experiments were performed at 30°C.

B "1 – cumulative probability" distribution functions of microtubule lengths at each initiation moment (black) and of the initiation probabilities of DDB runs in the presence of EB1 and Lis1 (blue). Conditions as shown in Fig 5G. 381 DDB complexes were analysed from two different experiments.

 © 2017 The Francis Crick Institute Limited
