# Supplementary material for: Combinatorial regulation of the balance between dynein microtubule end accumulation and initiation of directed motility
Source: EMBO J. 2017 Oct 16;36(22):3387–404. doi: 10.15252/embj.201797077 (PMC5686545; doi:10.15252/embj.201797077)
Supplement: Supplementary file 2 — Movie EV1 [file EMBJ-36-3387-s002.zip › Movie_EV1/Movie_EV1.docx]

**Movie EV1.** Microtubule plus-end tracking of GFP-dynein (green) localising to the plus ends of dynamic Alexa568-microtubules (magenta) in the presence of dynactin and EB1. Experimental condition as in Fig. 1A.
