# Supplementary material for: Combinatorial regulation of the balance between dynein microtubule end accumulation and initiation of directed motility
Source: EMBO J. 2017 Oct 16;36(22):3387–404. doi: 10.15252/embj.201797077 (PMC5686545; doi:10.15252/embj.201797077)
Supplement: Supplementary file 3 — Movie EV2 [file EMBJ-36-3387-s003.zip › Movie_EV2/Movie_EV2.docx]

**Movie EV2.** GFP-dynein (green) on dynamic Atto565-microtubules (magenta) in the presence of all DDB components and EB1, showing DDB motion and reduced end-tracking of GFP- dynein. Experimental condition as in Fig. 2D.
