# Supplementary material for: Combinatorial regulation of the balance between dynein microtubule end accumulation and initiation of directed motility
Source: EMBO J. 2017 Oct 16;36(22):3387–404. doi: 10.15252/embj.201797077 (PMC5686545; doi:10.15252/embj.201797077)
Supplement: Supplementary file 4 — Movie EV3 [file EMBJ-36-3387-s004.zip › Movie_EV3/Movie_EV3.docx]

**Movie EV3.** Dynactin-mediated GFP-dynein (green) plus end tracking on Atto565-microtubules (magenta) at an elevated dynactin concentration. Experimental condition as in Fig. 4A.
