# Supplementary material for: Combinatorial regulation of the balance between dynein microtubule end accumulation and initiation of directed motility
Source: EMBO J. 2017 Oct 16;36(22):3387–404. doi: 10.15252/embj.201797077 (PMC5686545; doi:10.15252/embj.201797077)
Supplement: Supplementary file 5 — Movie EV4 [file EMBJ-36-3387-s005.zip › Movie_EV4/Movie_EV4.docx]

**Movie EV4.** p150-mediated GFP-dynein (green) plus end tracking on Atto565-microtubules (magenta). Experimental condition as in Fig. 4B.
