# Supplementary material for: Combinatorial regulation of the balance between dynein microtubule end accumulation and initiation of directed motility
Source: EMBO J. 2017 Oct 16;36(22):3387–404. doi: 10.15252/embj.201797077 (PMC5686545; doi:10.15252/embj.201797077)
Supplement: Supplementary file 7 — Movie EV6 [file EMBJ-36-3387-s007.zip › Movie_EV6/Movie_EV6.docx]

**Movie EV6.** GFP-dynein (green) on dynamic Atto647N-microtubules (magenta) in the presence of all DDB components, EB1, and 5 μM mCherry-Lis1, showing GFP-dynein tracking plus- ends. Experimental condition as in Fig. 5C.
