# Supplementary material for: Combinatorial regulation of the balance between dynein microtubule end accumulation and initiation of directed motility
Source: EMBO J. 2017 Oct 16;36(22):3387–404. doi: 10.15252/embj.201797077 (PMC5686545; doi:10.15252/embj.201797077)

Fig EV1Ai. Hela cell dynactin

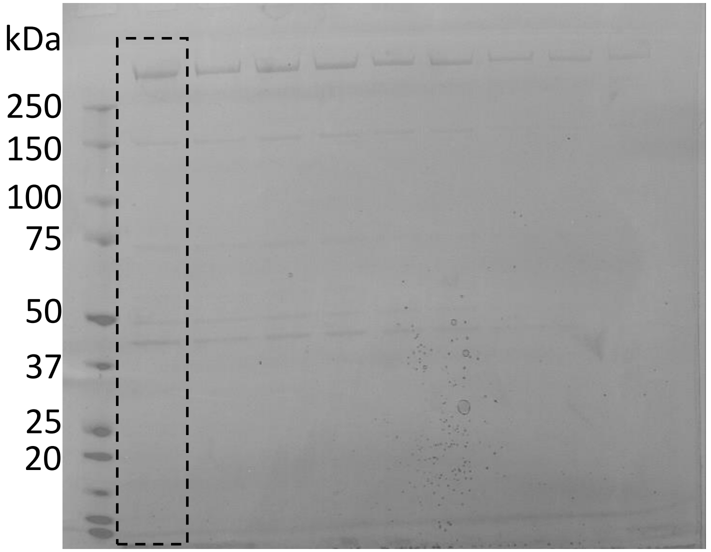

Fig EV1Aii. Hela cell dynactin

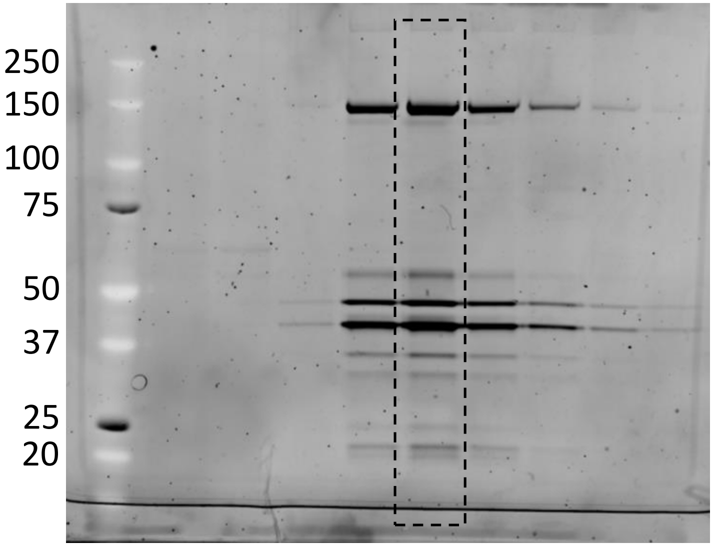

Fig EV1B. Pig dynactin

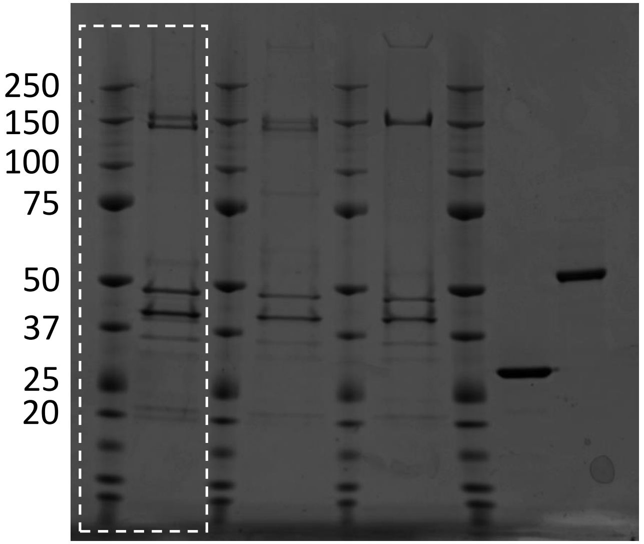

Fig EV1B. GFP dynein

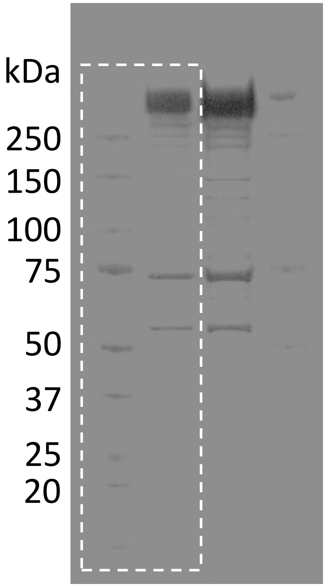

Fig EV1B. EB1/EB3

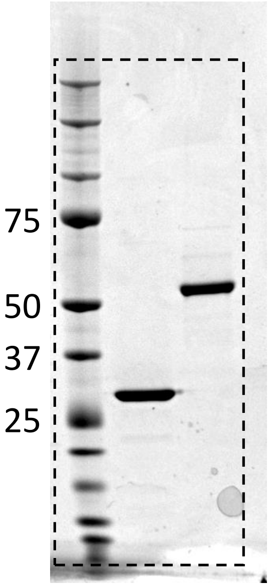

Fig EV1B. BicD2

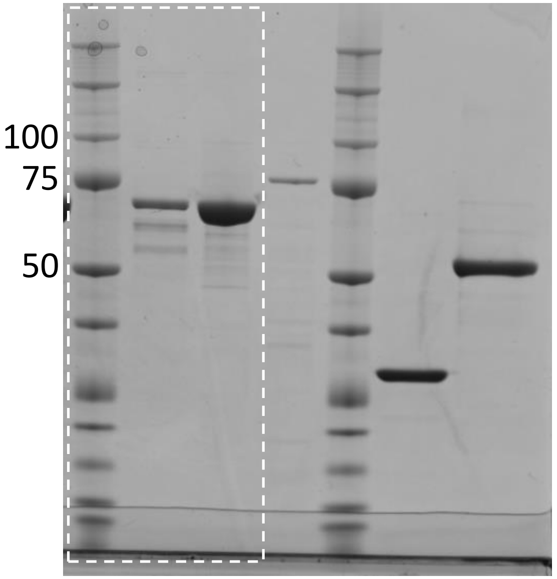

Fig EV1B. Lis1

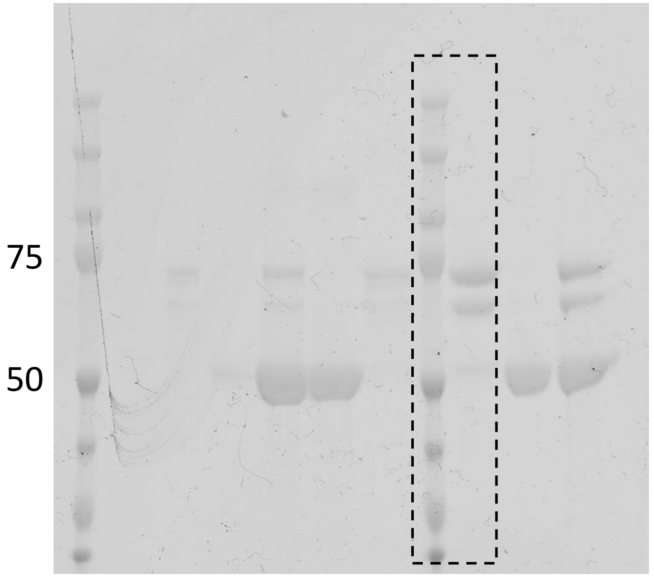

Supplement: Supplementary file 8 — Source Data for Expanded View [file EMBJ-36-3387-s008.zip › embj201797077-sup-0008-SDataFigEV1.pdf]
